# Supplementary figures and images for: Research on anchor chain visualization for a ship anchoring simulation training system
Source: PLoS One. 2020 Oct 6;15(10):e0237563. doi: 10.1371/journal.pone.0237563 (PMC7537884; doi:10.1371/journal.pone.0237563)

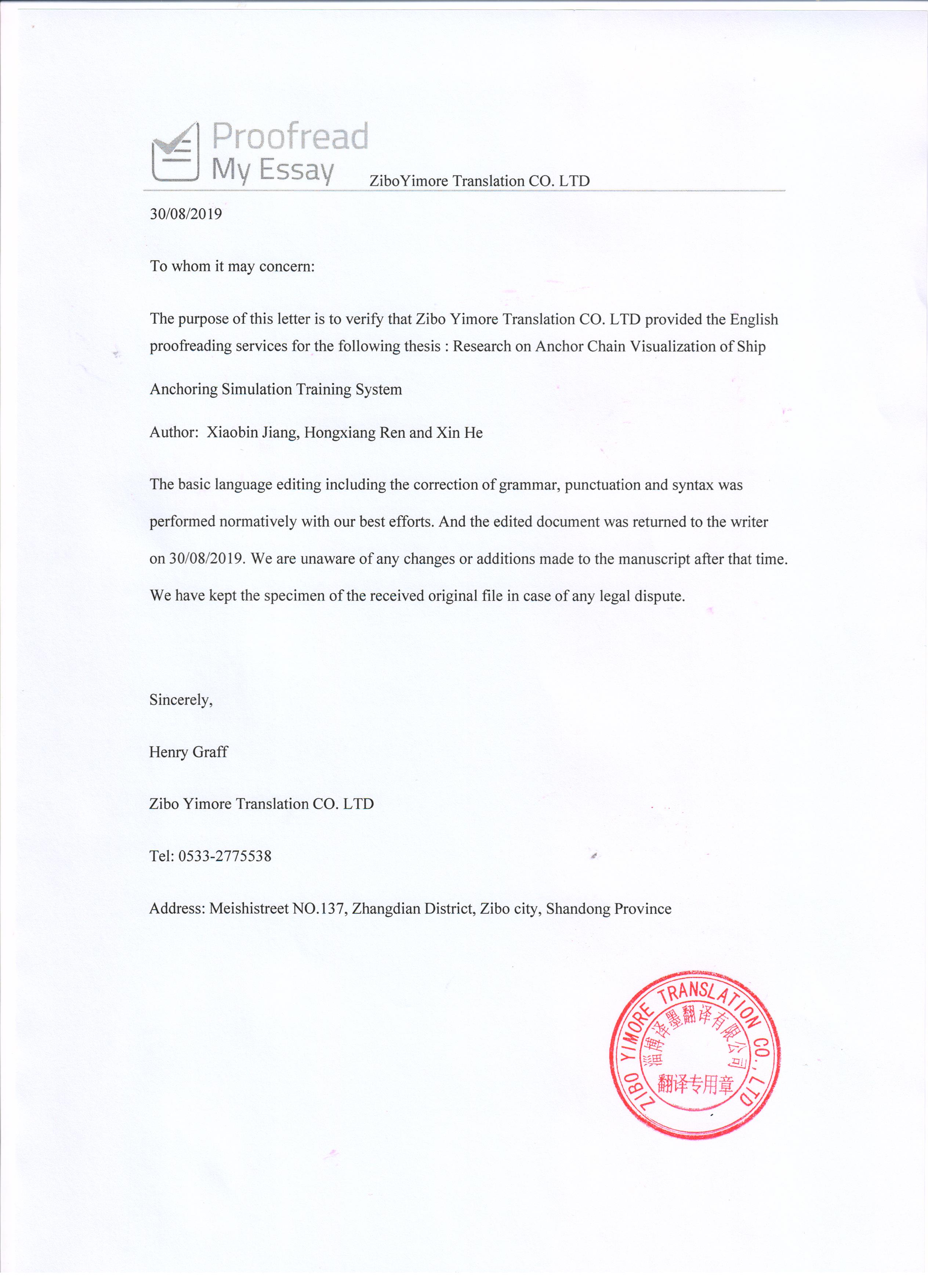

Supplement: S1 Fig — (JPG) [file pone.0237563.s001.jpg]
