# Supplementary material for: Research on anchor chain visualization for a ship anchoring simulation training system
Source: PLoS One. 2020 Oct 6;15(10):e0237563. doi: 10.1371/journal.pone.0237563 (PMC7537884; doi:10.1371/journal.pone.0237563)
Supplement: S1 Graphical abstract — (PDF) [file pone.0237563.s003.pdf]

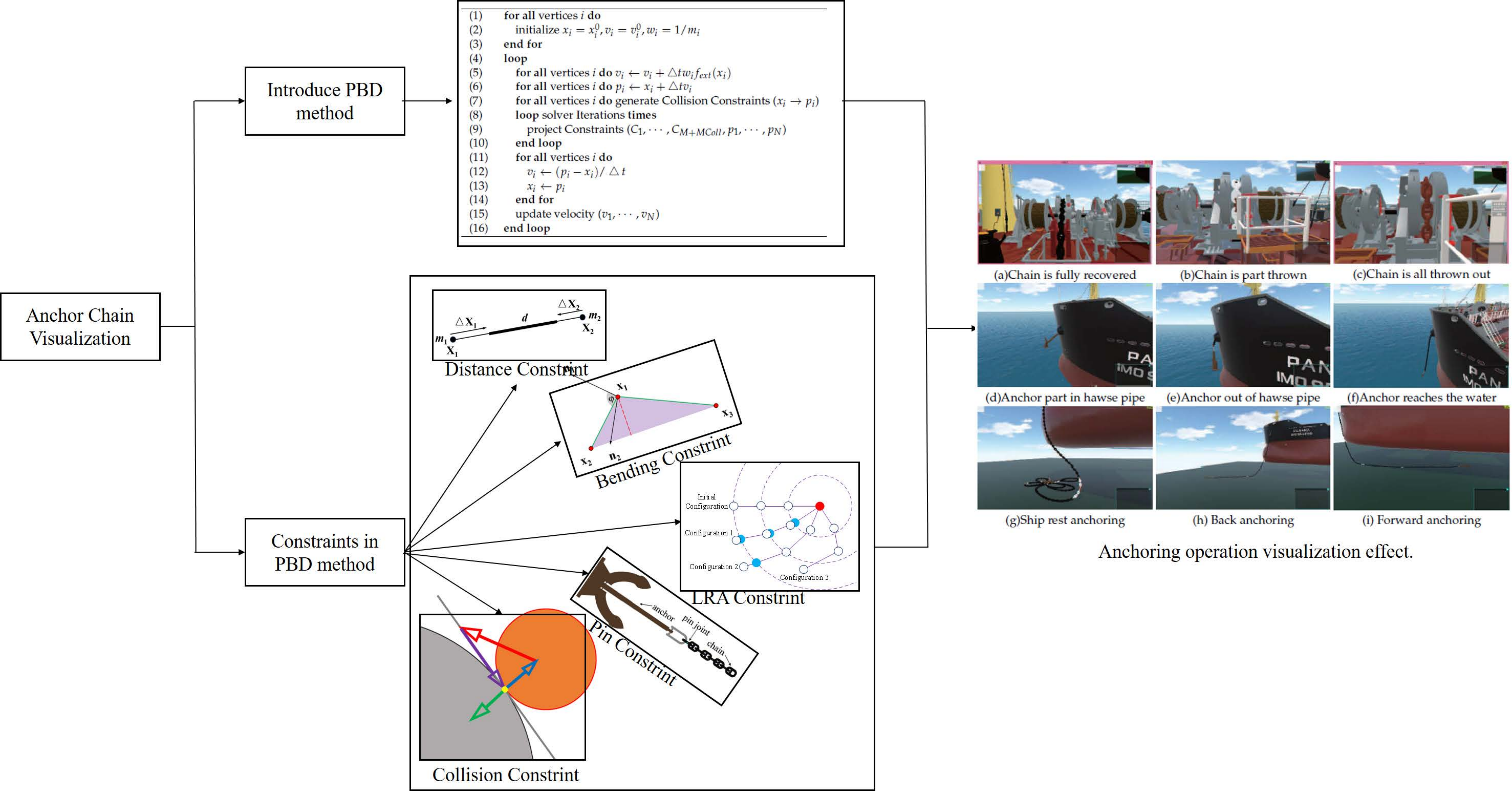
Anchor Chain Visualization
Introduce PBD method
for all vertices i do
initialize $x_i = x_i^0, v_i = v_i^0, w_i = 1/m_i$
end for
loop
for all vertices i do $v_i \leftarrow v_i + \triangle t w_i f_{ext}(x_i)$
for all vertices i do $p_i \leftarrow x_i + \triangle t v_i$
for all vertices i do generate Collision Constraints $(x_i \rightarrow p_i)$
loop solver Iterations times
project Constraints $(C_1, \cdots, C_{M+MColl}, p_1, \cdots, p_N)$
end loop
for all vertices i do
$v_i \leftarrow (p_i - x_i)/\triangle t$
$x_i \leftarrow p_i$
end for
update velocity $(v_1, \cdots, v_N)$
end loop
Constraints in PBD method
Distance Constrint
Bending Constrint
LRA Constrint
Initial Configuration
Configuration 1
Configuration 2
Configuration 3
Pin Constrint
anchor
pin joint
chain
Collision Constrint
(a)Chain is fully recovered
(b)Chain is part thrown
(c)Chain is all thrown out
(d)Anchor part in hawse pipe
(e)Anchor out of hawse pipe
(f)Anchor reaches the water
(g)Ship rest anchoring
(h) Back anchoring
(i) Forward anchoring
Anchoring operation visualization effect.
