# Supplementary material for: Research on anchor chain visualization for a ship anchoring simulation training system
Source: PLoS One. 2020 Oct 6;15(10):e0237563. doi: 10.1371/journal.pone.0237563 (PMC7537884; doi:10.1371/journal.pone.0237563)
Supplement: Attachment — Submitted filename: PONE-D-19-25283_comment.pdf [file pone.0237563.s004.pdf]

[Overall comment]

C0. Please, check grammar by a native speaker. There are lots of grammar mistakes.

[Introduction]

C1. Please, check grammar. For example, "Anchoring refer to…" should be "Anchoring refers to…".

C2. Please, change the sentence as follows

- ⇨ The difference phases of the anchor chain operation lead to…

C3. The author should read more carefully all of the sentences. The sentence should have 'subject' and 'verb'. For example, "To enhance the reality during ship anchoring. Combine with the development of the ship handling simulation system. Analyzes the movement posture of chain during anchoring."

[Related Work]

C4. The author split the implementation methods into static and dynamic analysis method. However, Mass-spring is also dynamic analysis method, not static. Could you suggest any other Mass-Spring method = Static?

C5. "the simulator is easy to <u>be implemented</u>."

C6. As far as I know, lumped mass method is to use spring between the mass. What is the main difference between the mass spring method and lumped mass method?

C7. "to replace the ship handling … " ➔ "to replace the anchor handling …"

C8. What is the differences between physical reality and behavioral reality?

C9. What is the main difference between related works and this paper? At the end of this section, you should discuss the originality of your research compared to the others.

[Position-Based Dynamics]

C10. The subscript in the equations should be changed. For example,

$$C_j(x_i 1, \cdots, x_i n j) = 0. \rightarrow C_j\left(x_{i_1}, \cdots, x_{i_{n_j}}\right) = 0$$

C11. Please, erase one of these two sentences.

Lastly, the corrected positions pi is applied to update the positions 75<br>and the velocities. Lastly, the corrected positions pi are used to update velocities and 76<br>positions.

C12. The caption of algorithm1 should be changed.

Position-based dynamic => Position-based dynamics

C13. You explained that "Substituting Eq (3) into Eq (2), λ is solved and then substituted back into Eq (3)." However, it is not easy to obtain eq (4) and (5) from eq (2) and (3). Please, explain how to obtain eq (4) and (5) in detail.

C14. Please, check the guide line of this journal about the chapter and sub-chapter. There is no numbers at each chapter. But you add 1 and 1.1 as a sub-chapter. Then, chapter 2, and chapter 3 is introduced. I think that Introduction should be "1. Introduction". Please, check the configuration of your paper.

[1.3 Long Range Attachment Constrain]

C15. The title should be "Long Range Attachment Constraint".

C16. You should explain more about Fig5. In the paragraph above Fig 5., you explained LRA method

by example. However, I'm not sure what will happen configuration 1, 2 and 3. Please, explain what will happen at each configuration.

[1.4 Pin Constraint]

C17. Please, don't add the explanation right after the caption. Please, move that sentences next to Fig 6,7,8,13 above or below paragraph.

[2. Discussion]

C18. I'm very confused because Fig.6 showed the real chain shape but Fig.9 shows only particles. Did you model the chain by using particles? The anchor should be model by chain. The realistic modeling of chains is the key of the anchor chain simulation.

C19. How about the friction? Did you consider the friction between chain and the hawsepipe or bell mouth, when pulling the chain?

C20. The discussion is the most important chapter in most of the papers. Your discussion is too short. I think you can discuss the following topics.

- Real time simulation: Compare the computing time according to the number of particles and constraints
- Anchor chain operation method: How to operation in the developed simulator
- Tendency of motion according to anchor chain hoisting speed.

[3. Conclusion]

C21. Please, suggest future research topic and direction for the readers.
