# Supplementary material for: Research on anchor chain visualization for a ship anchoring simulation training system
Source: PLoS One. 2020 Oct 6;15(10):e0237563. doi: 10.1371/journal.pone.0237563 (PMC7537884; doi:10.1371/journal.pone.0237563)
Supplement: Attachment — Submitted filename: Response to Editor.docx [file pone.0237563.s005.docx]

1. * Thank you for your response, and for letting us know the authors are the copyright owners.

Before we proceed, please explain in detail what software/tools were used to create the following:

Figs 2, 3, 6, 7, 8, 9, 12, 13, the supporting information file "chain.wmv," and the figure file "Graphical Abstract.pdf."

**Answer：**We use the program development software employee **Unity3D** and **Visual Studio 2015** to create the Figs 2, 3, 6, 7, 8, 9, 12, 13, the supporting information file "chain.wmv," and the figure file "Graphical Abstract.pdf."

2. We suggest you thoroughly copyedit your manuscript for language usage, spelling, and grammar. If you do not know anyone who can help you do this, you may wish to consider employing a professional scientific editing service.

Answer: We use professional scientific editing service by American Journal Experts (AJE) of my manuscript. We visit the AJE website (http://learn.aje.com/plos/), then we carefully check and update the grammar one more time before submitting.
