# Supplementary material for: Research on anchor chain visualization for a ship anchoring simulation training system
Source: PLoS One. 2020 Oct 6;15(10):e0237563. doi: 10.1371/journal.pone.0237563 (PMC7537884; doi:10.1371/journal.pone.0237563)
Supplement: Attachment — Submitted filename: Response to Reviewers.docx [file pone.0237563.s006.docx]

**Original Manuscript ID:** PONE-D-19-25283

**Original Article Title: “**Research on anchor chain visualization of ship anchoring simulation training system”

Dear Editor,

Thank you for allowing a resubmission of our manuscript, with an opportunity to address the reviewers’ comments.

We are uploading (a) our point-by-point response to the comments (below) (response to reviewers), (b) an updated manuscript with yellow highlighting indicating changes, and (c) a clean updated manuscript without highlights (PDF main document).

Best regards,

<Xiaobin Jiang> et al.

**Reviewer #1:**

**1. Reviewers' comments:** C0. Please, check grammar by a native speaker. There are lots of grammar mistakes.

**Responses to Questions:** Thank you for your nice comments on our article. We use professional scientific editing service by American Journal Experts (AJE) of my manuscript. We visit the AJE website (http://learn.aje.com/plos/), then we carefully check and update the grammar one more time before resubmitting.

**2. Reviewers' comments:** C1. Please, check grammar. For example, “Anchoring refer to…” should be “Anchoring refers to…”

**Responses to Questions:** Thanks for your careful checks. Based on your comments, we correct the grammar within the whole manuscript and change the sentence.

**3. Reviewers' comments:** C2. Please, change the sentence as follows

→The difference phases of the anchor chain operation lead to…

**Responses to Questions:** Thank you for your reminding. We have changed the sentence as “The different phases of operating the anchor chain lead to the complexity in simulating the chain.”

**4. Reviewers' comments:** C3. The author should read more carefully all of the sentences. The sentence should have ‘subject’ and ’verb’. For example, “To enhance the reality during ship anchoring. Combine with the development of the ship handling simulation system. Analyzes the movement posture of chain during anchoring.”

**Responses to Questions:** According to the reviewer’s comments, we have revised the manuscript extensively. We have changed the sentence as “To enhance reality during ship anchoring, we analyze the movement posture of the chain during anchoring, and then develop the ship handling simulation system.”

**5. Reviewers' comments:** C4. the author split the implementation methods into static and dynamic analysis method. However, mass-spring is also dynamic method, not static. Could you suggest any other mass-spring method = static?

**Responses to Questions:**  It is really a giant mistake to the whole quality of our article. We feel sorry for our mistake. The mass-spring method is dynamic method, not static. We have corrected it in the manuscript and we also feel great thanks for your point out.

The catenary method is extensively applied in the classification society [5]. The static analysis method is simple but not accurate. For dynamic analysis methods, there are numerous well-built methods for ship anchoring systems (e.g., the mass-spring method [6], the lumped mass method [7], the finite segment method [8], the finite element method [9], and the finite differences method [10]).

**6. Reviewers' comments:**  C5. “the simulator is easy to be implemented.”

Responses to Questions: Thank you for your reminding. We have erased the sentence and changed the formulation as “The static analysis method is simple but not accurate. For dynamic analysis methods, there are numerous well-built methods for ship anchoring systems (e.g., the mass-spring method [6], the lumped mass method [7], the finite segment method [8], the finite element method [9], and the finite differences method [10]).”

**7. Reviewers' comments:** C6. As far as I know, lumped mass method is to use spring between the mass. What is the main difference between the mass spring method and lumped mass method?

**Responses to Questions:** Mass spring method and lumped mass method are all use mass-less spring between the mass points.

Mass spring method divides the spring into three different types: structural spring, shear spring and bending spring, which respectively correspond to the calculation of structural force, shear force and bending force inside the chain. It is mainly used in the simulation visualization of cloth and rope. The calculated forces and moments are few, and the accuracy requirements are not high.

Lumped mass method needs to calculate many explicit forces and moments, and assume that all the forces are acting on the points. The calculation of tension is more accurate, and the solution is more time-consuming, the method mainly used in actual engineering requirements.

**8. Reviewers' comments:** C7. “to replace the ship handling…”→“to replace the anchor handling…”

**Responses to Questions:** Thank you for your reminding. We have changed the sentence“to replace the ship handling…” into “to replace the anchor handling…”.

**9. Reviewers' comments:** C8. What is the differences between physical reality and behavioral reality?

**Responses to Questions:** Physical reality is that when operate the simulator system, they are similar to the real equipment. The simulator should be equipped with operation equipment and corresponding response. Behavioral reality mainly depends on the control response of the participants, and the mathematical model of the simulation system. All in all, Physical reality is that physical devices are consistent with reality, Behavioral reality refers to the consistency between mathematical simulation and practice.

**10. Reviewers' comments:** C9. What is the main difference between related works and this paper? At the end of this section, you should discuss the origin of your research compared to the others.

**Responses to Questions:** The main difference between related works and this paper is that in the previous work PBD method is to realize the simulation of rope, cloth, deformable body, etc. It is rarely applied to the actual scene. In the ship anchoring operation, it is relatively difficult to simulate the retraction of anchor chain. In this paper, PBD method is applied to ship simulation. Through the research, we have improved the effect of the simulation of retraction and retraction of anchor chain. At the end of this section, we add discuss the origin of our research compared to the others. Introduces in the paper as follows:

PBD method is to realize the simulation of rope, cloth, deformable body, etc. It is rarely applied to the actual scene. In the ship anchoring operation, it is relatively difficult to simulate the retraction of anchor chain. In the paper, we use PBD method to simulate the process of chain release and retraction, and finally realize the three-dimensional visualization of mooring cable retraction and release, and finally develop the ship anchoring operation simulation system combining with the established three-dimensional ship.

**11. Reviewers' comments:** C10. The subscript in the equations should be changed. For example,

$$C_{j}(x_{i}1,\cdots,x_{i}n)=0\to C_{j}(x_{i_{1}},\cdots,x_{i_{n_{j}}})=0$$

**Responses to Questions:** According to the reviewers’ comments, we have changed the subscript in the equations. as $C_{j}(x_{i}1,\cdots,x_{i}n)=0\to C_{j}(x_{i_{1}},\cdots,x_{i_{n_{j}}})=0$

**12. Reviewers' comments:** C11. Please, erase one of these two sentences.

Lastly, the corrected positions pi is applied to update the positions 75

and the velocities. Lastly, the corrected positions pi are used to update velocities and 76 positions.

**Responses to Questions:** thanks for your careful checks. We are sorry for our carelessness. Based on your comments, we have erased one of the two sentences.

**13. Reviewers' comments:** C12. The caption of algorithm1 should be changed.

Position-based dynamic => Position-based dynamics

**Responses to Questions:** According to your suggestion, we have corrected the “Position-based dynamic” into “Position-based dynamics”.

**14. Reviewers' comments:** C13. You explained that “Substituting Eq (3) into Eq (2), λ is solved and then substituted back into Eq (3).” However, it is not easy to obtain eq (4) and (5) from eq (2) and (3). Please, explain how to obtain eq (4) and (5) in detail.

**Responses to Questions:** Thank you for your positive comments and valuable suggestions to improve the quality of our manuscript. We have improve equations to introduce how to get the eq(4) and eq(5). The revised content is as follows:

**15. Reviewers' comments:** C14. Please, check the guide line of this journal about the chapter and sub-chapter. There is no numbers at each chapter. But you add 1 and 1.1 as a sub-chapter. Then, chapter 2, and chapter 3 is introduced. I think that Introduction should be “1. Introduction”. Please, check the configuration of your paper.

**Responses to Questions:** We were really sorry for our carelessness. Thank you for your reminding. We have checked the configuration of our paper, and delete numbers at each chapter.

**16. Reviewers' comments:** C15.The title should be “Long Range Attachment Constraint”.

**Responses to Questions:**  Thanks for your careful checks. We are sorry for our carelessness. Based on your comments, we have corrected as “Long Range Attachment Constraint”.

**17. Reviewers' comments:** C16. You should explain more about Fig5. In the paragraph above Fig 5., you explained LRA method by example. However, I’m not sure what will happen configuration 1,2 and 3. Please, explain what will happen at each configuration.

**Responses to Questions:** Your suggestion really means a lot to us. Yes, it would be more understandable if we explain more about Fig5. So, we explain the LRA constraint below:

The LRA method is shown in Fig. 5 to simulate an inextensible chain attached to a hawsehole (red point). Each chain particle is constrained into the hawsehole centered on the attachment point. The radius of the sphere is the initial distance from the particle to the attachment point. For each configuration, target chain positions are marked in blue when particles should be projected. The chain particles in the constraint spheres are allowed to move freely, and particles outside the constraint sphere need to be projected onto the sphere. For example, in configuration 1, the chain particles will move to the blue point, and in configuration 3, the chain particles will move freely.

**18. Reviewers' comments:** C17. Please, don ’t add the explanation right after the caption. Please, move that sentences next to Fig 6,7, 8, 13 above or below paragraph.

**Responses to Questions:** We really appreciate your help and patience. We have moved the explanation sentences right after the caption inside the paper.

**19. Reviewers' comments:** C18. I‘m very confused because Fig.6 showed the real chain shape but Fig.9 shows only particles. Did you model the chain by using particles? The anchor should be model by chain. The realistic modeling of chains is the key of the anchor chain simulation.

**Responses to Questions:** Yes, we model the chain by using particles. We model the chain through PBD method. The PBD method to simulate rope or chain used by particle. The anchor chain we have seen is three model created by 3ds Max, by using the established 3D anchor chain model to replace the particles in the PBD method, and then we can visualize the simulation of anchor chain.

**20. Reviewers' comments:** C19. How about the friction? Did you consider the friction between the chain and hawsepipe or bell mouth, when pulling the chain?

**Responses to Questions:** We consider the friction between the chain and hawsepipe or bell mouth, when pulling the chain. As said in the paper, once the objects contact each other, a collision response is generated. According to the material properties of the particles and the collision object, different collision responses are generated. The particle material properties cover friction, viscosity, etc. The particle material properties cover friction, viscosity, etc. As shown in Fig 10, the purple arrow refers to the friction force, removing a percentage of the tangential velocity component.

**21. Reviewers' comments:** C20. The discussion is the most important chapter in of papers. Your discussion is too short. I think you can discuss the following topics.

- Real time simulation: Compare the computing time according to number of particles and constraints

- Anchor chain operation method: How to operation in the developed simulator

- Tendency of motion according to anchor chain hoisting speed.

**Responses to Questions:** Thank you for your nice comments on our article. We don't have a lot of discussions, which need to be supplemented. At this stage, the purpose of the simulation system is to improve the real-time visualization effect while meeting the accuracy. We want to do discuss from your point of view later, now we are suffering from the epidemic of novel coronavirus pneumonia in China, and there is no computer nearby. If we want to do further in-depth research, I can only finish it after we go back to school.

**22. Reviewers' comments:** C21. Please, suggest future research topic and direction for the readers.

**Responses to Questions:**  According to your suggestion, we have suggested future research topic and direction for the readers. Detailed revision was shown as follows. However, there are also has some disadvantages. The elastic stiffness of the chain is very large, although the stiffness of the model can be reduced by LRA method, it cannot be completely removed. Decoupling these parameters as well as adaptive time stepping are problems and important topics for future work.

**Reviewer #2:**

**1. Reviewers' comments:** The manuscript needs editing to English grammar and sentence for clear understanding to the reader.

**Responses to Questions:** Thank you for your nice comments on our article. We use professional scientific editing service by American Journal Experts (AJE) of my manuscript. We visit the AJE website (http://learn.aje.com/plos/), then we carefully check and update the grammar one more time before submitting.

**2. Reviewers' comments:** What is the ship handling simulation system? It is mentioned that the system has 20 million surfaces. The study present position-based dynamics, that means it is based on the mesh. It needs more explanations.

**Responses to Questions:** The ship handling simulation system is a part of the ship handling simulator used to train the crew. The system is mainly used to model and simulate the anchoring operation in 3D ship, and to study the dynamic response of ship anchoring.

In order to increase the immersion of the simulation system, we have built a three-dimensional model of the whole ship in the simulation system. All these three-dimensional models have 20 million surfaces together. We use the PBD method to study the simulation the retraction and release of the anchor chain. The PBD method is based on the particle.

**3. Reviewers' comments:** p. 7. Line 129. What is the actual situation and how the chain can be specified like that?

**Responses to Questions:** We thank you for constructive criticism, and time spent to analyze this manuscript, the responses and explanations related to this comment as below:

The anchor chain has the characteristics of high tensile rigidity and high impact toughness, When the maximum bending amount is relatively low, the simulation effect is more in line with the actual situation of mooring line. We can specify the anchor chain with high bending resistance but low stretching stiffness to satisfy the actual situation.

**4. Reviewers' comments:** p. 10. Line 194. It is not easy to understand why the friction force should be removed.

**Responses to Questions:** We feel sorry that we did not explain clear about the sentence. We should say the purple arrow indicates the friction force applied to the particles, which eliminates a certain proportion of the tangential velocity.

**5. Reviewers' comments:** p.11. Line 225. How to define the x0? Is it related to time step?

**Responses to Questions:** *x*_0_ correspond to critical point of function g(*x*_0_). Indeed,▽*g*=0. That is to say, *x*_0_ is the value of minimum g(*x*). Total Frame Time = Iteration Time * Iteration Count + Collision Time. So *x*_0_ is related to time step.

**6. Reviewers' comments:** p.12. Line 246. It is mentioned that the seabed penetration problem is solved but the problem is not mentioned in the manuscript. If the problem can be solved with the environment collision, it should be discussed in the section.

**Responses to Questions:** We thank the reviewer for the kind comments. In the Environment Collision section, we have introduced the seabed penetration problem can be solved by the environment collision. The penetration problem of anchor chain and seabed is the same as that of anchor chain and other objects, so there is no special explanation for the penetration problem of seabed. Figure 13(g), (h) and (i) shows the situation of anchor chain lies on the seabed.

**7. Reviewers' comments:** The simulation speed and error between PBD method and Newton method in the discussion. However, the verification of the simulation result should be added in the discussion. It is necessary to show the comparison between the exact solution and the simulation result.

**Responses to Questions:** Thank you for your nice comments on our article. Our main purpose is to research for crew training, so the simulation system needs to achieve satisfactory visual effect with as little resource consumption as possible to meet the real-time requirements. Accurate results are difficult to obtain, in discussion part, we describe the results of iteration times and relative errors of the two methods, can be seen from figure 14. In 5 iterations the relative error is 9.02*10^-3^, which satisfy the simulation accuracy requirement, so it is not necessary to calculate in more iterations.

**//****************************************************************************//**

**//2020.05.29*******************************************************************//**

**//****************************************************************************//**

**1.** **Reviewers' comments:** * Thank you for your response, and for letting us know the authors are the copyright owners.

Before we proceed, please explain in detail what software/tools were used to create the following:

Figs 2, 3, 6, 7, 8, 9, 12, 13, the supporting information file "chain.wmv," and the figure file "Graphical Abstract.pdf."

**Answer：**We use the program development software employee **Unity3D** and **Visual Studio 2015** to create the Figs 2, 3, 6, 7, 8, 9, 12, 13, the supporting information file "chain.wmv," and the figure file "Graphical Abstract.pdf."

**2. Reviewers' comments:** We suggest you thoroughly copyedit your manuscript for language usage, spelling, and grammar. If you do not know anyone who can help you do this, you may wish to consider employing a professional scientific editing service.

**Responses to Questions:** We use professional scientific editing service by American Journal Experts (AJE) of my manuscript. We visit the AJE website (http://learn.aje.com/plos/), then we carefully check and update the grammar one more time before submitting.

//****************************************************************************//

//2020.05.05*******************************************************************//

//****************************************************************************//

**1. Reviewers' comments:** Before we can proceed with your manuscript, we ask that you please clarify the following:

a) Please clarify if the software used to create the following images and video was developed by the authors of this study: Figs 2, 3, 6, 7, 8, 9, 12, 13, the supporting information file "chain.wmv," and the figure file "Graphical Abstract.pdf."

Please also confirm these figures and supporting information file have not been published before.

**Responses to Questions:** The following images and video was developed by the authors of this study: Figs 2, 3, 6, 7, 8, 9, 12, 13, the supporting information file "chain.wmv," and the figure file "Graphical Abstract.pdf."

We also confirm that these figures and supporting information file have not been published before.

b) If the authors of this study did not create the software themselves, please explain where the authors obtained the images for Figs 2, 3, 6, 7, 8, 9, 12, the supporting information file "chain.wmv," and the Figure file "Graphical Abstract.pdf" in your submission.

**Responses to Questions:** Figures 2, 3, 6, 7, 8, 9, 12, the supporting information file "chain.wmv," and the Figure file "Graphical Abstract.pdf" in our submission are all developed by ourselves.

c) Please state whether Figs 2, 3, 6, 7, 8, 9, 12, 13, the supporting information file "chain.wmv," and the Figure file "Graphical Abstract.pdf" have been previously copyrighted.

**Responses to Questions:** Figs 2, 3, 6, 7, 8, 9, 12, 13, the supporting information file "chain.wmv," and the Figure file "Graphical Abstract.pdf" have not been published before.

d) If any of the images in the above mentioned figures and video have been previously copyrighted, PLOS ONE is unable to publish these, as all content is published under the Creative Commons Attribution (CC BY) 4.0 license, which means that they will be freely available online, and any third party is permitted to access, download, copy, distribute, and use these materials in any way, even commercially, with proper attribution.

**Responses to Questions:** No images in the above mentioned figures and video have been previously copyrighted.

We require one of the following:

i) Specific consent from the copyright holder to publish these images in PLOS ONE, under the CC BY 4.0 license. Please include a file including the granted permission as a Supporting Information file and include the following text within your figure caption(s): “Republished from [ref] under a CC BY license, with permission from [name of publisher], original copyright [original copyright year].”

ii) The authors remove these images from their submission.

**Responses to Questions:** The copyright owner is ourselves, and the author of this article will license PlosOne to publish.

**2. Reviewers' comments:** We note that your manuscript is not formatted using one of PLOS ONE’s accepted file types. Please reattach your manuscript as one of the following file types: .doc, .docx, .rtf, or .tex (accompanied by a .pdf).

If your submission was prepared in LaTex, please submit your manuscript file in PDF format and attach your .tex file as “other.”

**Responses to Questions:** We will send our manuscript to you in .tex (accompanied by a .pdf). The thesis we submitted is prepared in latex and submitted in PDF format. When submitting, we will attach the .tex file as "other".

**3. Reviewers' comments:** Please amend your Response to Reviewers letter to include a point by point response to each of the points made by the Editor and / or Reviewers. Please follow this link for more information: http://blogs.PLOS.org/everyone/2011/05/10/how-to-submit-your-revised-manuscript/

**Responses to Questions:** We amend our Response to Reviewers letter to include a point by point response to each of the points made by the Editor and / or Reviewers.

**4. Reviewers' comments:** We suggest you thoroughly copyedit your manuscript for language usage, spelling, and grammar. If you do not know anyone who can help you do this, you may wish to consider employing a professional scientific editing service.

**Responses to Questions:** We use professional scientific editing service by American Journal Experts (AJE) of my manuscript. We visit the AJE website (http://learn.aje.com/plos/), then we carefully check and update the grammar one more time before submitting.

Upon resubmission, please provide the following:

The name of the colleague or the details of the professional service that edited your manuscript

**Answer:** The names of colleagues editing the manuscript include Hongxiang Ren, Xin He, etc. The professional services of editing manuscripts are “Zibo Yimore Translation CO.LTD” editing company.

A copy of your manuscript showing your changes by either highlighting them or using track changes (uploaded as a *supporting information* file)

Answer: We uploaded a high lighting manuscript as a *supporting information* file.

A clean copy of the edited manuscript (uploaded as the new *manuscript* file)

Answer: We uploaded a clean copy of the edited manuscript as the new *manuscript* file.
